# Emodin Exerts Dual Hepatoprotective/Hepatotoxic Effects Dependent on Metabolic Microenvironment via Gut–Liver Axis Crosstalk: A Multi-Omics Study in MAFLD and Normal Mice

**DOI:** 10.3390/ijms27104411

**Published:** 2026-05-15

**Authors:** Rui Mao, Yuxia Guo, Haiyan Zhang, Ruiqi Wang, Yangang Cheng, Huifeng Li, Yan Wang, Yingli Wang

**Affiliations:** College of Tradition Chinese Medicine, Shanxi University of Chinese Medicine, Jinzhong 030619, China; mr_0517@163.com (R.M.);

**Keywords:** MASLD, emodin, dose-response relationship, host metabolic microenvironment, gut-liver axis, multi-omics integration

## Abstract

Emodin exhibits promising hepatomodulatory potential, yet its precise actions remain poorly characterized across distinct hepatic pathological states. Dose-dependent effects of emodin (40, 80, and 120 mg/kg) were investigated in mice with metabolic dysfunction-associated steatotic liver disease (MASLD), as well as healthy control mice, through integrated histopathological, biochemical, metabolomic, and gut microbiota profiling. The 80 mg/kg dose tended to yield the most favorable hepatoprotective outcomes in MASLD mice, whereas the 120 mg/kg dose was accompanied by apparent adverse hepatic alterations in normal mice. Correlative analyses further suggested that such divergent hepatic responses may be potentially linked to the gut microbiota–short-chain fatty acid (SCFA) axis, as well as the modulation of BAX/Bcl-2 balance and ESR1 expression. The hepatic effects of emodin are state- and dose-dependent. The dose of 80 mg/kg may represent a plausible candidate therapeutic range for MASLD, and the gut–liver axis is proposed as a likely underlying regulatory pathway.

## 1. Introduction

Metabolic dysfunction-associated steatotic liver disease (MASLD) has emerged as the most prevalent chronic liver disorder globally [1,2]. Its high prevalence is closely linked to underlying metabolic abnormalities, and it is rapidly surpassing viral hepatitis as the leading cause of hepatic morbidity. MASLD carries a significant risk of progression from simple steatosis to steatohepatitis, fibrosis, and ultimately hepatocellular carcinoma. Moreover, its prognosis is strongly associated with cardiovascular disease—the primary cause of mortality in this population—highlighting its role as a key hepatic manifestation of systemic metabolic dysregulation. Clinically, MASLD management faces dual challenges: the reliance on imaging and invasive liver biopsy for diagnosis due to a lack of convenient non-invasive biomarkers, and the absence of effective pharmacological therapies beyond lifestyle modification [3,4,5]. Thus, investigating novel mechanisms, particularly those involving the gut–liver axis, is crucial for developing new diagnostic tools and treatments targeting gut microbiota and related pathways [6,7,8].

Dysregulation of the gut–liver axis represents a core pathological mechanism in MASLD onset and progression. This bidirectional communication network, encompassing the biliary tract, portal venous system, and systemic circulation, plays a vital role in maintaining intestinal homeostasis while modulating hepatic metabolism and immune responses. In MASLD, gut–liver axis disruption is characterized by intestinal dysbiosis, impaired barrier integrity, and increased hepatic exposure to gut-derived metabolites, collectively driving steatosis, inflammation, and fibrosis [9,10].

Alterations in gut microbiota composition are an early event in this process [9]. Patients with MASLD typically exhibit reduced microbial diversity, with an increased abundance of pathogenic taxa (e.g., Enterobacteriaceae) and decreased levels of beneficial symbionts (e.g., Bacteroides, Akkermansia) [11,12]. These shifts disrupt metabolic output, elevating harmful metabolites such as endogenous ethanol and lipopolysaccharides (LPS), while reducing beneficial products like short-chain fatty acids (SCFAs) and bile acids. Concurrently, impairment of the intestinal barrier—through mucus layer thinning, downregulation of tight-junction proteins, and increased vascular permeability—facilitates bacterial and metabolite translocation into the portal circulation, thereby exacerbating liver injury [13,14].

Emodin, a natural anthraquinone derived from traditional herbal medicine, exhibits diverse pharmacological activities, including anti-inflammatory, lipid-modulating, antimicrobial, and antioxidant effects [15,16,17]. In hepatic contexts, emodin has demonstrated potential in non-alcoholic fatty liver disease (NAFLD) models by attenuating lipid synthesis via AMPK/PPARγ signaling and suppressing inflammation and fibrosis through modulation of MAPK/NF-κB pathways [18,19,20,21,22].

Nevertheless, critical knowledge gaps remain. The dynamic equilibrium underlying emodin’s effects of hepatoprotection versus hepatotoxicity lacks mechanistic clarity. Furthermore, a well-defined dose–response relationship and a safe therapeutic window have not been established. Most mechanistic studies have focused narrowly on hepatic pathways, with limited investigation into the compound’s systems-level actions across the gut–liver axis–microbiota–metabolite network.

This study aims to systematically elucidate the differential hepatic effects of emodin in MASLD versus healthy mice and to characterize its dose-dependent properties. From a gut–liver axis perspective, we will investigate its explanatory mechanisms, focusing on how emodin dose-dependently reshapes gut microbiota composition and short-chain fatty acid (SCFA) metabolism, thereby modulating key gut–liver signaling pathways to exert hepatoprotective effects in MASLD mice versus potential hepatotoxic outcomes in normal mice.

## 2. Results

### 2.1. Histological Observation of Liver and Colon Tissues

**Figure 1:**
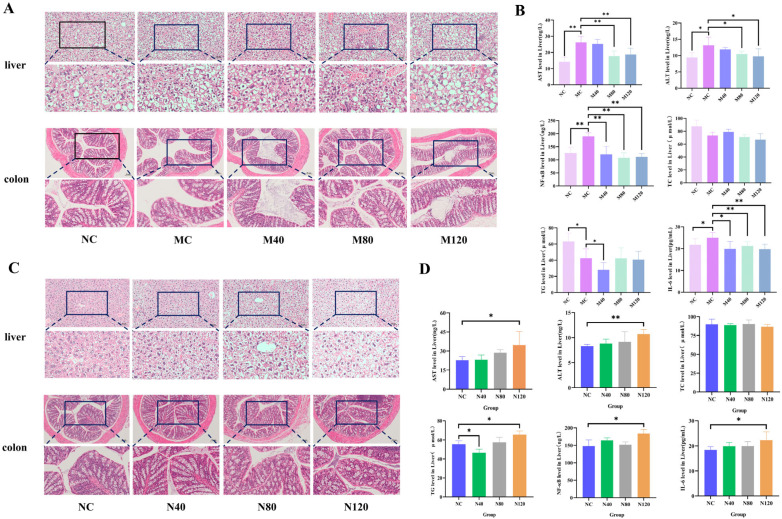
Pathological changes and comparison of serum AST, ALT, IL-6, NF-κB, TC and TG levels. (**A**) HE staining results of liver and colon tissues in the MASLD group. (**B**) Levels of AST, ALT, IL-6, NF-κB, TC and TG in serum of the MASLD group. (**C**) HE staining results of liver and colon tissues in the Sham group. (**D**) Levels of AST, ALT, IL-6, NF-κB, TC and TG in serum of the sham group, *n* = 6, * *p* < 0.05, ** *p* < 0.01). All samples were collected after 12 h of fasting at the end of emodin intervention, and all indicators were measured at this time point. Data were analyzed by one-way ANOVA followed by Dunnett’s multiple comparison test, and are presented as mean ± SD.

As shown in Figure 1A,C, HE staining of liver tissues and colon tissues revealed the following morphological changes.

In the MASLD group, the model control (MC) group exhibited hepatocellular steatosis, hydropic degeneration, and lobular inflammation. The MASLD + emodin 40 mg/kg (M40) and MASLD + emodin 80 mg/kg (M80) groups showed gradual restoration of hepatocellular morphology and reduced inflammation, whereas the MASLD + emodin 40 mg/kg (M120) group displayed mild steatosis. The MC group showed shortened crypts and inflammatory cell infiltration in the colon tissue. The M40 group showed no significant changes, while the M80 group exhibited intact crypt morphology and reduced inflammation. In contrast, the M120 group showed significant crypt loss.

In the sham group, the normal control (NC) group demonstrated hepatocytes with large, round, and centrally located nuclei, intact morphology, and tightly arranged structures. The NC + emodin 40 mg/kg (N40) group showed no significant changes compared to the NC. The NC + emodin 80 mg/kg (N80) group exhibited mild hydropic degeneration in a small number of hepatocytes (characterized by intracellular water accumulation and swelling). In contrast, the NC + emodin 120 mg/kg (N120) group displayed extensive ballooning degeneration, along with focal eosinophilic necrosis and focal lytic necrosis—i.e., apoptotic bodies—scattered throughout the hepatic lobules, which represents a hallmark pathological feature. The NC group displayed intact colonic epithelium with tightly connected and regularly arranged epithelial cells, well-structured crypts with normal goblet cells, and no inflammatory infiltration. The N40 group showed no obvious changes. The N80 group exhibited thinning of the muscular layer, loosely connected epithelial cells, gland atrophy, disorganized arrangement, and crypt shortening. The N120 group showed crypt branching, significantly shortened crypt length, and inflammatory cell infiltration.

### 2.2. Tissues Biochemical Assays

As shown in Figure 1B, in the MASLD group, the levels of alanine aminotransferase (ALT), aspartate aminotransferase (AST), interleukin-6 (IL-6), and nuclear factor-κB (NF-κB) were significantly elevated in the MC group compared with the NC group. Both the M80 and M120 treatments markedly suppressed these increases, with a more pronounced effect observed in the M80 group. In the sham group (Figure 1D), ALT and AST levels in the N40 and N80 groups showed no significant difference relative to the NC group. However, the N120 group exhibited significantly increased ALT (*p* < 0.01) and AST (*p* < 0.05), indicating that continuous administration of high-dose emodin for 56 days could induce liver injury. Total cholesterol (TC) measurements revealed that emodin had no significant effect on TC levels in liver and colon tissues. In contrast, emodin regulated triglyceride (TG) levels in a dose-dependent manner: compared with the NC group, TG content was significantly decreased in the N40 group (*p* < 0.05), but markedly increased in the N120 group (*p* < 0.05). Meanwhile, high-dose emodin (N120 group) significantly upregulated the levels of IL-6 and NF-κB in liver and colon tissues (*p* < 0.05), suggesting that 56-day continuous administration of high-dose emodin may trigger an inflammatory response in these tissues.

### 2.3. Metabolomic Analysis

#### 2.3.1. Metabolomic Analysis of Liver Tissue and Colon Tissue Based on UHPLC/Q-Orbitrap-MS

**Figure 2:**
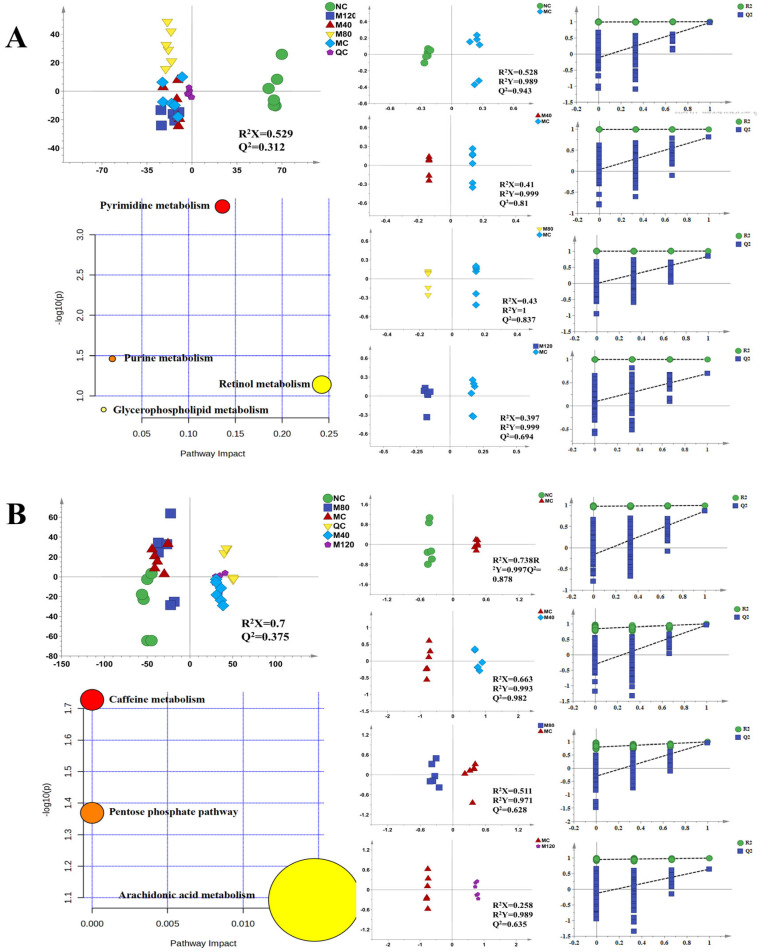
Metabolomic analysis of liver and colon tissues in the MASLD group ((**A**) liver tissue; (**B**) colon tissue, *n* = 6). Liver and colon tissues were harvested immediately after the last emodin administration for subsequent metabolomic profiling.

**Figure 3:**
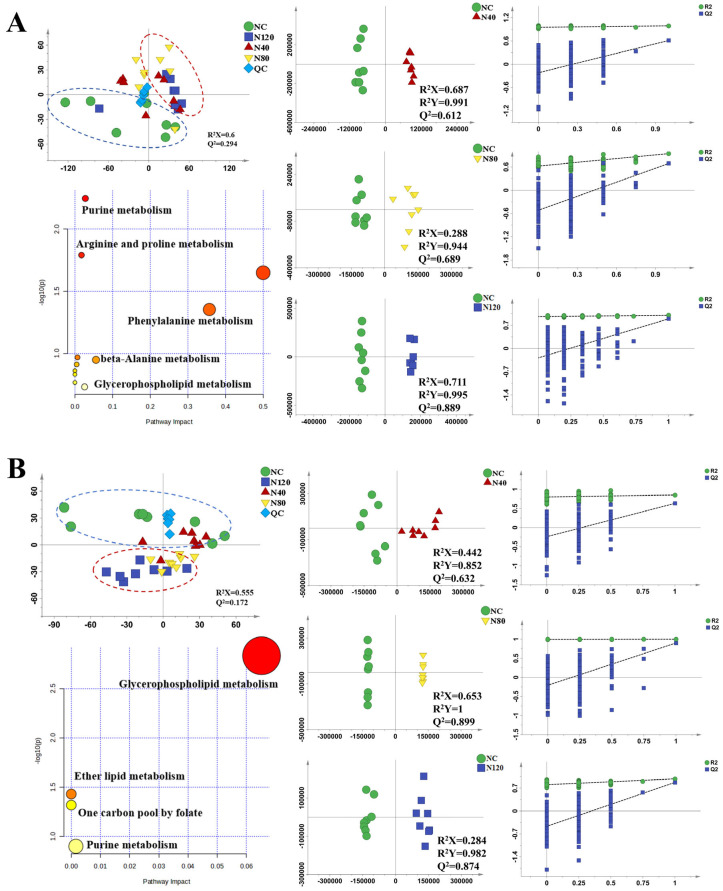
Metabolomic analysis of liver and colon tissues in the sham group ((**A**) liver tissue; (**B**) colon tissue, *n* = 6). Liver and colon tissues were harvested immediately after the last emodin administration for subsequent metabolomic profiling.

To investigate whether administration of emodin affects liver and intestinal metabolism in MASLD mice and normal mice, we conducted metabolomic analyses of liver and colon tissues (Figure 2 and Figure 3). In the MASLD group, the preliminary analysis and clustering of the principal component analysis (PCA) model reveal that the MC group and M40 group did not exhibit significant separation, whereas both were clearly separated from the NC group, M80 group, and M120 group. In the sham group, the NC group and the N40 group did not show clear separation, whereas both were distinctly separated from the N80 and N120 groups. This indicates that different doses of emodin altered metabolic profiles in the liver and colon of MASLD mice and normal mice.

An orthogonal partial least squares discriminant analysis (OPLS-DA) model was established to analyze the difference in liver metabolism in mice before and after administration of emodin. The results of the OPLS-DA analysis exhibited clear separation. Differential metabolites were screened based on a variable importance in projection (VIP) > 1 and *p* < 0.05 in the OPLS-DA models. Furthermore, differential metabolites were identified based on retention time, molecular weight, ion type, and a mass error tolerance of ±10 ppm using databases including PubChem, LipidMAPS, HMDB, and Xcalibur. In the MASLD group, a total of 22 common differential metabolites regulated by the MC group and different emodin doses were identified in liver tissue (Table S1), and 16 were identified in colon tissue (Table S2). In the sham group, a total of 17 common differential metabolites regulated by the NC group and different doses of emodin were identified in liver tissue (Table S3), and 21 were identified in colon tissue (Table S4).

#### 2.3.2. Metabolic Pathway Enrichment Analysis

These metabolites were imported into the MetaboAnalyst 5.0 database for metabolic pathway analysis to further explore the potential mechanisms by which different doses of emodin affect the liver and colon tissues of MASLD mice and sham mice. Enrichment analysis in the MASLD group showed four significantly altered metabolic pathways in liver tissue: glycerophospholipid metabolism, pyrimidine metabolism, purine metabolism, and retinol metabolism, among which glycerophospholipid metabolism was the most significant. In colon tissue, the major affected pathways included caffeine metabolism, the pentose phosphate pathway, and arachidonic acid metabolism. In the liver tissue of the sham group, five related metabolic pathways were identified: glycerophospholipid metabolism, ether lipid metabolism, folate-mediated one-carbon metabolism, and purine metabolism, with glycerophospholipid metabolism being the most significantly enriched. In colon tissue, the biosynthesis of phenylalanine, tyrosine, and tryptophan was identified as the most significantly altered pathway.

### 2.4. Effects of Emodin on the Gut Microbiota in Mice

#### 2.4.1. Effects of Emodin on Alpha and Beta Diversity of Gut Microbiota

**Figure 4:**
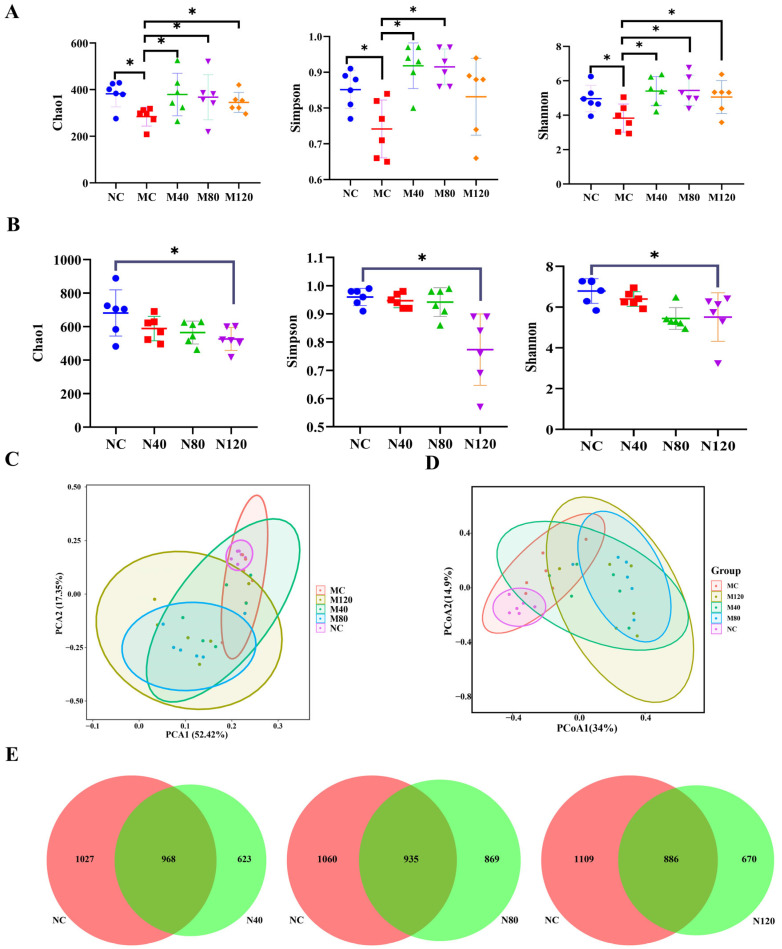
Effects of emodin on alpha diversity and beta diversity of gut microbiota. (**A**) Effects of emodin on gut microbiota alpha diversity in MASLD mice (*n* = 6). (**B**) Effects of emodin on gut microbiota alpha diversity in Sham mice (*n* = 6). (**C**,**D**) Effects of emodin on gut microbiota beta diversity in MASLD mice (*n* = 6). (**E**) Effects of emodin on gut microbiota beta diversity in Sham mice (*n* = 6). * *p* < 0.05. Colonic contents were harvested immediately after the last emodin administration for 16S rRNA sequencing and diversity analysis. Data were analyzed by one-way ANOVA followed by Dunnett’s multiple comparison test, and are presented as mean ± SD.

The Chao1 index is used to estimate species richness in a sample; the higher the value, the more potential species are present in the gut microbiota. The Shannon and Simpson diversity indices comprehensively assess microbial diversity by considering both the number of species and their evenness. The higher the values of these indices, the greater the diversity. As shown in Figure 4A, in the MASLD group, the MC group showed significantly reduced Chao1, Simpson, and Shannon indices compared to the NC group, which were significantly reversed after emodin administration. As shown in Figure 4B, in the sham group, emodin treatment significantly decreased the Chao1 index, and the Simpson and Shannon indices also showed decreasing trends. These results indicate that emodin administration reduced the quantity, richness, diversity, and relative abundance of the gut microbiota in mice.

Beta diversity analysis reflects differences in microbial community composition between groups, typically using multivariate statistical methods such as PCA and principal coordinates analysis (PCoA).

The beta diversity analyses of the MASLD group outcomes are depicted in Figure 4C,D. On PCA2, the NC group was closely grouped, while the MC group displayed an upward deviation, making them distinguishable from the NC group. The M80 group shows a significant separation trend compared to the MC group (Figure 4C). In Figure 4D, along the PCoA1 axis, the M80 group shows a significant separation trend compared to the MC group. The beta diversity analysis of sham group outcomes is depicted in Figure 4E, and as the dosage of emodin increases (N40, N80, and N120), fewer gut bacteria overlap with the NC group.

#### 2.4.2. Effects of Emodin on Gut Microbial Composition

**Figure 5:**
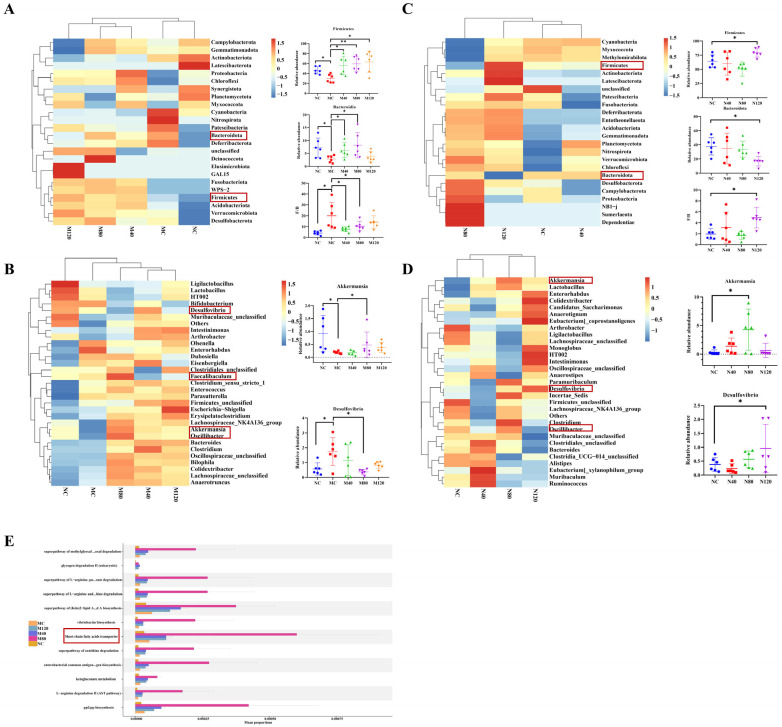
Effects of emodin on the relative abundance of gut microbiota in the MASLD group and Sham group ((**A**,**C**) phylum level; (**B**,**D**) genus level; (**E**) Functional Prediction STAMP Differential Analysis, *n* = 6, * *p* < 0.05, and ** *p* < 0.01). Colonic contents were harvested immediately after the last emodin administration for 16S rRNA sequencing and diversity analysis. Data were analyzed using one-way ANOVA followed by Dunnett’s multiple comparison test and are presented as the mean ± SD.

To illustrate the effects of emodin on the gut microbiota composition of the MASLD group and the sham group, the top 30 species at the phylum level were compared. At the phylum level (Figure 5A,C), in the MASLD group, the relative abundances of Firmicutes and Bacteroidetes were significantly reduced in the MC group. These changes were notably reversed in the M80 group. As shown in Figure 5A, the F/B ratio in the MC group was significantly increased (*p* < 0.05). This increase was reversed in the M40 and M80 groups, but significantly increased again in the M120 group compared with the NC group (*p* < 0.05). In the sham group, the abundance of Firmicutes increased while that of Bacteroidetes decreased in the N120 group compared to the NC group. The F/B ratio of the N120 group was significantly higher than that of the NC group (*p* < 0.05).

At the genus level (Figure 5B,D), compared with the NC group, the MASLD group’s MC group showed significant changes in the genera Desulfovibrio and Akkermansia, while the M80 group significantly reversed these changes (*p* < 0.05). In sham group, compared with the NC group, Akkermansia was significantly increased in the N80 group (*p* < 0.05), whereas Desulfovibrio was significantly increased in the N120 group (*p* < 0.05).

#### 2.4.3. PICRUSt2 Function Prediction

As shown in Figure 5E, based on the functional prediction results of PICRUSt2, the M80 group is significantly enriched in pathways related to “short-chain fatty acid synthesis”.

In summary, emodin influences the gut microbiota in a dose-dependent manner, and its effects differ depending on the hepatic status of mice. In the MASLD model mice, medium-dose emodin (80 mg/kg) may help improve the gut microbial structure, while in sham mice, high-dose emodin may exert a certain adverse impact on gut microbial homeostasis.

### 2.5. Different Doses of Emodin on SCFA Metabolites in Mice

**Figure 6:**
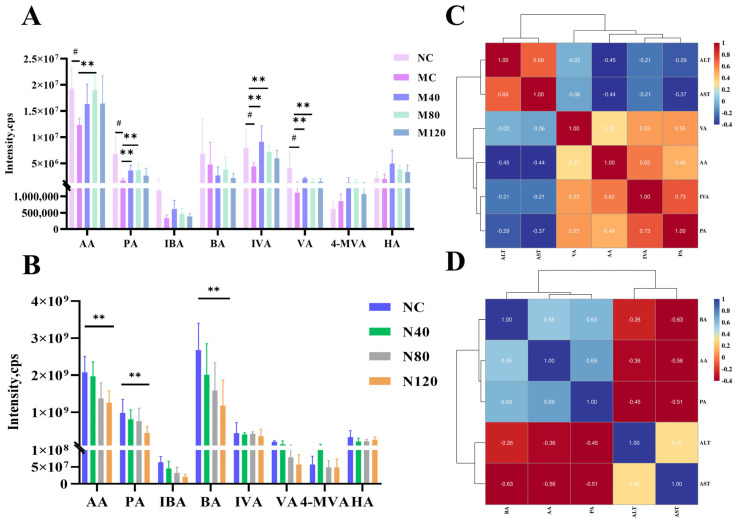
Dose-dependent changes in short-chain fatty acid levels ((**A**) Bar chart of short-chain fatty acid levels in colon contents in the MASLD group; (**B**) Bar chart of short-chain fatty acid levels in colon contents in the Sham group; (**C**) Correlation analysis between short-chain fatty acid levels and liver function indicators in the MASLD group; (**D**) Correlation analysis between short-chain fatty acid levels and liver function indicators in the Sham group. *n* = 6, # *p* < 0.05 NC vs. MC group, ** *p* < 0.01). Colonic contents were harvested immediately after the last emodin administration for short-chain fatty acid quantification. Data were analyzed using one-way ANOVA followed by Dunnett’s multiple comparison test and are presented as the mean ± SD.

Short-chain fatty acids (SCFAs) in mouse colon contents were qualitatively analyzed using gas chromatography–mass spectrometry (GC–MS). Standard solutions and cecal content samples from each group were analyzed, including: acetic acid (AA), propionic acid (PA), isobutyric acid (IBA), butyric acid (BA), isovaleric acid (IVA), valeric acid (VA), 2-ethylbutyric acid (internal standard), 3-methylvaleric acid, 4-methylvaleric acid (4-MVA), and hexanoic acid (HA). In the MASLD group (Figure 6A), compared with the NC group, the MC group showed significantly reduced levels of AA, PA, IVA, and VA (all ** *p* < 0.01). Both the M80 and M120 emodin treatment groups significantly increased these SCFA levels, with the M80 group showing a more pronounced recovery effect. In the sham group (Figure 6B), high-dose emodin (N120) significantly decreased the levels of AA and BA compared with the NC group (** *p* < 0.01), indicating that long-term high-dose emodin administration may impair the production of beneficial SCFAs in the gut. Correlation analysis (Figure 6C,D) revealed that in the MASLD group, the liver injury markers ALT and AST were significantly positively correlated (*r* = 0.69). A synergistic variation trend was observed among short-chain fatty acids (PA and IVA, *r* = 0.73). Critically, ALT/AST showed moderate negative correlations with acetic acid (AA) (*r* = −0.45/−0.44), suggesting that the gut microbiota metabolite acetic acid may be inversely associated with the degree of liver injury in the MASLD model. Correlation analysis in the sham group showed significant positive correlations among the major short-chain fatty acids (BA, AA, and PA), indicating a synergistic trend in their changes in this model. Critically, these short-chain fatty acids exhibited varying degrees of negative correlations with the liver injury markers ALT and AST, with the strongest negative correlation observed between butyric acid (BA) and AST (*r* = −0.63). These findings further support the potential protective effect of gut microbiota-derived short-chain fatty acids against liver injury in the MASLD model.

### 2.6. Network Toxicological Analysis of Emodin

**Figure 7:**
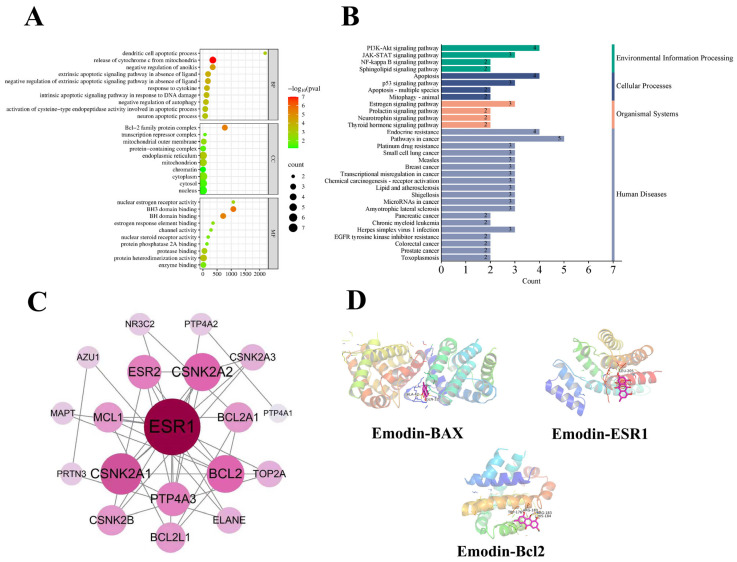
Network toxicological analysis of emodin ((**A**) GO functional enrichment analysis; (**B**) KEGG enrichment analysis; (**C**) Core targets of emodin-induced liver injury; (**D**) Molecular docking diagram).

Toxicity analysis using AMDETlab revealed that the highest probability of adverse effects was eye irritation (probability: 0.961), followed by drug-induced liver injury (DILI, probability: 0.935). A total of 34 emodin-related targets were identified through the ChEMBL, SwissTargetPrediction, and SEA databases. Meanwhile, 1801 liver injury-associated targets were screened using the Genecards, TTD, and OMIM databases. The intersection of these two sets yielded nine potential targets linked to emodin-induced liver injury. GO functional enrichment analysis, encompassing biological processes (BP), cellular components (CC), and molecular functions (MF), is illustrated in Figure 7A. BP primarily involves the regulation of hormones, neurons, and apoptosis. CC is mainly associated with BCL2 family protein complexes, endoplasmic reticulum, and mitochondria. MF includes BH domain binding, protease binding, and nuclear estrogen receptor activity. KEGG pathway analysis identified key pathways related to apoptosis, the PI3K-Akt signaling pathway, and endocrine resistance (Figure 7B). PPI network and core target analysis revealed that the core targets associated with emodin-induced liver injury include ESR1, BCL2, and others (Figure 7C).

### 2.7. Molecular Docking

Using emodin as the ligand, molecular docking was performed with core targets (ESR1, BCL2) and the apoptosis-related protein BAX as receptors. The results indicated that emodin exhibited strong molecular binding affinity with ESR1, BCL2, and BAX (see Table 1). The molecular docking interactions between emodin and the three targets were visualized using PyMOL 2.5 software (Figure 7D).

### 2.8. Western Blot Analysis

**Figure 8:**
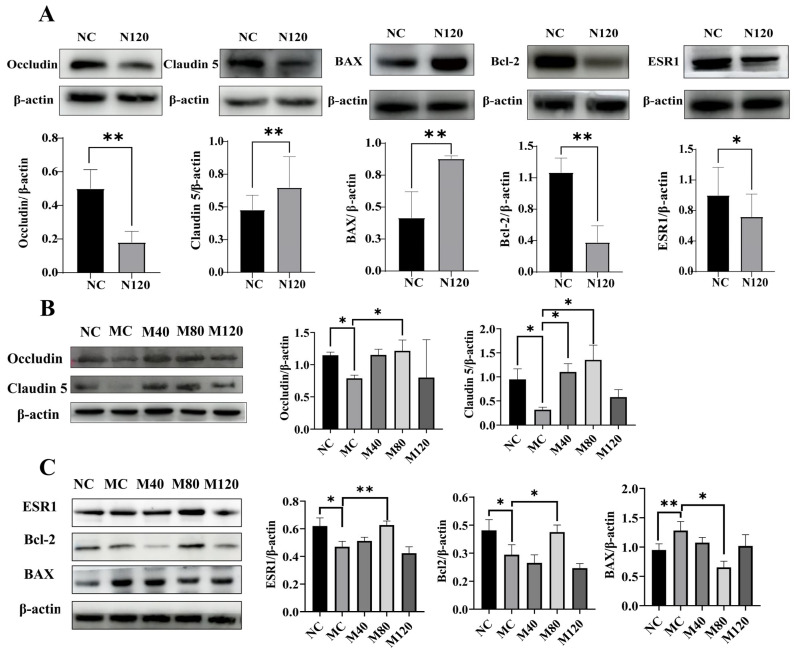
The protein expression of Occludin and Claudin 5 in the colon of mice, as well as the protein expression of BAX, BCL2 and ESR1 in the liver of mice. (**A**) Protein expression of mice treated with high-dose emodin in the Sham group; (**B**,**C**) Protein expression of mice treated with emodin in the MASLD group. *n* = 6, * *p* < 0.05, and ** *p* < 0.01. All colon and liver tissue samples were collected after 12 h of fasting at the end of emodin intervention, and protein expression levels were measured by Western blot at this time point. Data were analyzed using one-way ANOVA followed by Dunnett’s multiple comparison test, and are presented as the mean ± SD.

To explore the dose-dependent effects of emodin on the gut–liver axis and intestinal barrier, Western blot was used to detect the protein levels of key tight junction markers (Occludin, Claudin-5) in colonic tissues, as well as the expression profiles of ESR1, BCL2, and BAX in hepatic tissues, providing preliminary correlative evidence for the predictions derived from network toxicology analysis. In the MASLD MC group, Occludin and Claudin-5 were downregulated (Figure 8B), while BAX and ESR1 were upregulated (Figure 8C). Emodin treatment (M40, M80, and M120) reversed these changes. Notably, compared with the M40 and M80 groups, the high-dose group (M120) showed a downward trend in the levels of Occludin and Claudin-5 (Figure 8B). Furthermore, in the sham group, high-dose emodin significantly decreased colonic Occludin (*p* < 0.01) and Claudin-5 (*p* < 0.01), reduced hepatic BCL2 (*p* < 0.05) and ESR1 (*p* < 0.05), but increased BAX (*p* < 0.01) expression versus the control (Figure 8A).

### 2.9. Integration Analysis

**Figure 9:**
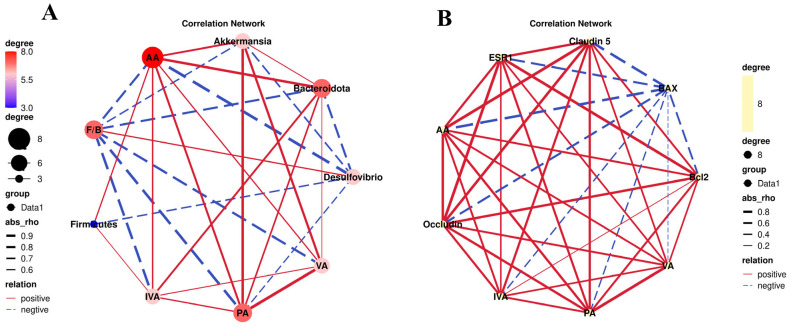
Correlation analysis between gut microbiota, SCFAs, and the liver–gut axis pathway. (**A**) Correlation analysis between levels of SCFAs (AA, VA, IVA, and PA) and gut microbiota; (**B**) Correlation analysis between the levels of SCFAs and protein expression (Occludin, Claudin-5, ESR1, BCL2, and BAX).

Spearman correlation analysis was used in this study to evaluate the associations among differential gut microbiota, SCFAs, and key molecules involved in the gut–liver axis. In the microbiota–SCFA correlation network (Figure 9A), Akkermansia and Bacteroidota showed strong positive correlations with AA, VA, and PA (*p* < 0.05); Firmicutes were positively correlated with acetic acid (AA) and isovaleric acid (IVA) (*p* < 0.05). In contrast, Desulfovibrio exhibited significant negative correlations with AA and PA (*p* < 0.05). In the SCFA–gut–liver axis molecular correlation network (Figure 9B), AA, VA, IVA, and PA were strongly positively correlated with the tight junction proteins Occludin and Claudin-5, as well as the anti-apoptotic markers ESR1 and BCL2 (*p* < 0.05); meanwhile, these SCFAs displayed significant negative correlations with the pro-apoptotic marker BAX (*p* < 0.05).

## 3. Discussion

Despite their widespread clinical use, the hepatotoxic effects of most medicinal plants remain unevaluated, and they are often presumed to be safe. In contrast, emodin—a key bioactive component of rhubarb (*Rheum* spp.) and Polygonum multiflorum—has been implicated in hepatotoxicity by growing evidence, prompting considerable concern. It was found that low-to-moderate doses (40–80 mg/kg) of emodin conferred significant therapeutic benefits in the MASLD model. However, at a high dose (120 mg/kg), these benefits were attenuated in diseased mice, and marked hepatointestinal toxicity was induced in normal mice. This bidirectional, dose-dependent effect demonstrates that the pharmacological action of emodin is state-dependent, being determined by the host’s underlying pathophysiological condition.

Emodin-induced liver injury has been frequently reported [23,24,25,26]. However, some studies have indicated that drug-induced liver injury is unlikely to be the sole cause of elevated liver enzymes. There is a direct correlation between systemic inflammation (characterized by the levels of IL-6, C-reactive protein, and ferritin) and liver injury. In particular, there is a direct association between IL-6 production and elevated AST levels [27]. Furthermore, studies have confirmed that emodin can inhibit splenocyte proliferation triggered by excessive inflammation. It restores immune balance by reducing the levels of pro-inflammatory cytokines such as TNF-α and IL-6, while increasing the level of the anti-inflammatory cytokine IL-10 [28]. Meanwhile, this immunomodulatory effect is closely associated with the progression of MASLD [29,30,31]. Immune cells, fibroblasts, endothelial cells, and hepatocytes regulate the production of IL-6, thereby coordinating the acute-phase response of the liver [32]. Emodin is a free anthraquinone, and its main absorption site is the small intestine. Therefore, this study explored the effects of emodin on mouse liver tissue based on the gut–liver axis.

Based on histological and biochemical data, in the MASLD mouse model, a dose of 80 mg/kg of emodin improved hepatic steatosis, inflammation, and serum transaminase levels, while also enhancing colonic structure; however, at a dose of 120 mg/kg, the recovery of colonic function was inhibited. In contrast, high doses of emodin in sham-treated mice induced significant hepatocyte injury and impaired colonic integrity.

UHPLC/Q-Orbitrap-MS-based metabolomic analysis revealed that emodin dose-dependently reshaped hepatic and colonic metabolic profiles in both MASLD and normal mice. Principal component analysis (PCA) indicated distinct clustering. Pathway enrichment analysis showed that the liver of MASLD mice exhibited significant alterations in glycerophospholipid, pyrimidine, purine, and retinol metabolism, whereas the colon was primarily affected in caffeine metabolism, the pentose phosphate pathway, and arachidonic acid metabolism. In contrast, the liver of normal mice showed perturbations in glycerophospholipid, ether lipid, folate-mediated one-carbon, and purine metabolism, while the colon displayed marked changes in phenylalanine, tyrosine, and tryptophan biosynthesis. Phenylalanine, tyrosine, and tryptophan are important nutritional sources for colonic microorganisms [33], and their presence and content can influence the composition and structure of the colonic microbial community. Glycerophospholipids are the main components of the liver cell membrane, accounting for more than 50% of the membrane lipids. They form the basic framework of the cell membrane in the form of a phospholipid bilayer, providing a site for the embedding and attachment of membrane proteins, maintaining the fluidity and stability of the cell membrane, and ensuring the normal morphology and structure of liver cells. They are also the basis for physiological activities such as material exchange and signal transduction in cells. Additionally, glycerophospholipid metabolites can regulate the activation, proliferation, and cytokine secretion of immune cells, affecting the immune defense and immune surveillance functions of the liver [34].

The gut microbiota plays a crucial role in the intestinal ecosystem. Dysbiosis of the gut microbiota may participate in various attacks on the liver through the gut–liver axis. On one hand, over-activated immune cells induced by bacterial products may lead to liver injury, inflammation, and fibrosis, thereby accelerating the development of liver injury. On the other hand, metabolites from gut bacteria, such as SCFAs, can improve the inflammatory response, oxidative damage, and lipogenesis in liver tissue [35]. Gut microbiota analysis demonstrated that the impact of emodin was contingent upon both dosage and hepatic status. In MASLD mice, the MC group exhibited significantly reduced α-diversity (Chao1, Shannon, and Simpson) compared to the NC group. This decline was substantially reversed by the M80 intervention, which also improved β-diversity. At the phylum level, the elevated Firmicutes/Bacteroidota (F/B) ratio in the MC group was normalized in the M80 group but increased again in the M120 group. Genus-level analysis revealed that the M80 treatment effectively counteracted the aberrant shifts in Desulfovibrio and Akkermansia observed in the MC group. Functional prediction further indicated enrichment of the ‘short-chain fatty acid synthesis’ pathway in the M80 group. In contrast, in normal mice, the high dose (N120) reduced α-diversity, increased the F/B ratio, and elevated the abundance of Desulfovibrio. In summary, a moderate dose of emodin (80 mg/kg) restores a healthier gut microbiota architecture in MASLD, whereas a high dose disrupts microbial homeostasis in normal mice.

SCFAs are produced by various bacterial groups, including acetate (50–70%, synthesized by multiple bacteria), propionate (10–20%, synthesized by Bacteroidetes and certain Firmicutes), and butyrate (10–40%, produced by a few Clostridium species). They affect immune responses in the intestine and those related to the peripheral circulation and distal parts of the body [36,37]. Detecting SCFAs is essential when studying the gut–liver axis. When the gut–liver axis is imbalanced, changes in SCFA levels may affect the function of immune cells. GC-MS analysis of short-chain fatty acids (SCFAs) in colon contents revealed that the effect of emodin on SCFA levels depends on the hepatic condition of the mice. In MASLD mice, the M80 group showed an increasing trend in AA, PA, and BA levels. In contrast, in normal mice, a high dose of emodin (N120) significantly reduced the levels of AA, PA, and BA. Correlation analysis further demonstrated that the levels of AA, PA, and BA were negatively correlated with serum ALT and AST indices, suggesting that SCFAs may mediate hepatoprotective effects in this process.

Network toxicology prediction suggested the potential of emodin to induce drug-induced liver injury (DILI). Intersection analysis of databases identified nine potential liver injury-related targets. GO and KEGG enrichment analyses indicated that these targets were primarily involved in biological processes and pathways such as hormone regulation, apoptosis, and the PI3K-Akt signaling pathway. PPI network analysis pinpointed core targets including ESR1 and BCL2, and molecular docking confirmed strong binding affinity of emodin to ESR1, BCL2, and BAX.

The intestinal barrier is mainly composed of tightly connected adjacent cells, which are a group of proteins with related structures and functions. Among them, Claudin and Occludin play key roles. Occludin and Claudin-5 (a member of the Claudin family) play important roles in the gut–liver axis and can regulate barrier permeability. Experimental validation revealed that in MASLD mice, emodin intervention (M40, M80, and M120) upregulated the expression of colonic tight junction proteins (Occludin, Claudin-5), which were downregulated by the disease, and downregulated the elevated levels of BAX and ESR1. However, the restorative effect was attenuated at the high dose (M120). In normal mice, a high dose of emodin (N120) disrupted colonic tight junction integrity, significantly upregulated the hepatic pro-apoptotic protein BAX, and downregulated the anti-apoptotic protein BCL2 and ESR1.

Correlation analysis showed that the abundances of beneficial bacterial genera (Akkermansia, Bacteroidota) and SCFAs (including acetic acid, propionic acid, and valeric acid) were positively correlated with the expression levels of tight junction proteins BCL2 and ESR1, while negatively correlated with BAX expression. The synchronous alterations of these indicators preliminarily suggest a potential association among the gut microbiota–SCFA–ESR1/BCL2/BAX pathway. The existing literature has confirmed that microbiota-derived SCFAs can regulate the transcription and protein expression of ESR1 through epigenetic modification and receptor signaling pathways, exhibit estrogen receptor downregulatory activity, and serve as a crucial mediator in upstream signal regulation [38]. As a key nuclear receptor target, ESR1 mediates the expression balance of downstream apoptosis-related factors BCL2 and BAX via transcriptional regulation, thereby determining cell survival and apoptosis. This target axis has been recognized as a core regulatory module in the research of metabolic disorders and organ injury. Furthermore, screening core targets by network pharmacology combined with molecular docking and animal experiments to verify the expression profile of the ESR1/BCL2/BAX pathway further supports the scientific validity and rationality of the target selection and pathway hypothesis in this study [39].

The results of this study demonstrate a robust synergistic correlation between the gut microbiota composition, SCFA levels, and the expression of ESR1, BCL2, and BAX proteins, suggesting potential co-regulatory mechanisms among the gut microbiota, SCFAs, and ESR1/BCL2/BAX proteins. However, this study is limited to correlation analyses across multi-omics data, microbial metabolism, and protein expression, without conducting functional validation experiments such as SCFA supplementation, ESR1 intervention, or gene silencing. The precise causal regulatory mechanisms of this pathway require further investigation in dedicated subsequent studies.

It is worth noting that this study used methods such as network toxicology, molecular docking, and PICRUSt2 functional prediction to explore the potential mechanisms and regulatory pathways of emodin-induced hepatotoxicity. Although these methods provide valuable guidance for in vivo experimental validation, they all have inherent limitations: network toxicology relies on existing database information and may have incomplete target screening, and it can only predict potential associations without confirming the actual effects of targets in vivo or their dose-dependent impacts; molecular docking is based on a single protein crystal structure, does not simulate the complex in vivo environment, can only reflect binding capacity without confirming in vivo protein functional changes, and does not consider the in vivo metabolic transformation of emodin; and PICRUSt2 predicts microbial functional potential based on 16S rRNA gene sequencing data and related assumptions, which may be inaccurate, and it only predicts potential functions, without detecting actual functional gene expression or metabolite production, nor does it take into account host factors affecting microbial functional expression.

## 4. Materials and Methods

### 4.1. Reagents and Materials

Emodin (Batch No.: S30728-25 g) was purchased from Shanghai Yuanye Biotechnology Co., Ltd. (Shanghai, China). Sodium carboxymethylcellulose was obtained from Tianjin Bailingcen Biotechnology Co., Ltd. (Tianjin, China). Hematoxylin–eosin (H&E) staining solution (Lot No.: 20220425) was supplied by Fuzhou Feijing Biotechnology Co., Ltd. (Fuzhou, China). LC/MS-grade acetonitrile (Cat. No.: 215625) and methanol (Cat. No.: 216678) were products of Fisher Chemical (Pittsburgh, PA, USA). Formic acid (Cat. No.: H1913009) was purchased from Shanghai Aladdin Biotechnology Co., Ltd. (Shanghai, China). The methionine-choline-deficient (MCD) diet and methionine-choline-sufficient (MCS) control diet were both obtained from Beijing Sybef Biotechnology Co., Ltd. (Beijing, China).

Commercial ELISA kits for AST, ALT, TC, TG, IL-6, and NF-κB (Lot Nos.: F2856-A, F2260-A, F30043-A, F30053-A, F2163-A, and F2836-A) were all supplied by Shanghai Fankewi Technology Co., Ltd. (Shanghai, China), and used according to the manufacturer’s instructions.

Molecular biology reagents included Phusion^®^ Hot Start Flex 2× Master Mix (Lot No.: M0536L, Shanghai Yitao Biological Instrument Co., Ltd. (Shanghai, China)), DL2000 DNA Marker (Lot No.: 3427A, Takara Bio Inc. (Beijing, China)), GeneColor nucleic acid stain (Lot No.: GBY-1, Beijing Jinboyi Biotechnology Co., Ltd. (Beijing, China)), Qubit™ dsDNA HS Assay Kit (Lot No.: Q32854, Invitrogen, Life Technologies (Carlsbad, CA, USA)), Biowest Agarose G-10 (Lot No.: 111860, Biowest (Nuaillé, France)), 50× TAE buffer (Lot No.: B548101-500, Sangon Biotech (Shanghai) Co., Ltd. (Shanghai, China)), AMPure XT magnetic beads (Lot No.: A63880, Beckman Coulter (Brea, CA, USA)), and NovaSeq 6000 SP Reagent Kit (Lot No.: 20028402, Illumina (San Diego, CA, USA)).

Short-chain fatty acid standard compounds, including acetic acid (≥99.8%, Lot No.: C12700504), propionic acid (≥99.5%, Lot No.: C14458542), isobutyric acid (≥99.5%, Lot No.: C13706285), n-butyric acid (>99.5%, Lot No.: C14253081), isovaleric acid (≥99.0%, Lot No.: C14414245), and n-valeric acid (≥99.5%, Lot No.: C14026528), were purchased from Shanghai Macklin Biochemical Co., Ltd. (Shanghai, China). 2-Ethylbutyric acid (≥99.5%, Lot No.: H2231563) was obtained from Shanghai Aladdin Biotechnology Co., Ltd.

Antibodies and reagents for Western blot analysis were listed as follows: Claudin 5 (A10207) and Occludin (A2601) from ABclonal (Wuhan, China); BAX (50599-2-lg) and BCL2 (68103-1-lg) from Proteintech (Wuhan, China); ESR1 (54257-1) from SAB (Baltimore, MD, USA); β-actin (GB23301) from Wuhan Servicebio Technology Co., Ltd. (Wuhan, China); and HRP-conjugated goat anti-rabbit secondary antibody (GB23303) from Beijing Solarbio Science & Technology Co., Ltd. (Beijing, China). Protease inhibitor PMSF (CR2303056) and phosphorylated protease inhibitor cocktail (CR2307130) were purchased from Wuhan Servicebio Technology Co., Ltd. (Wuhan, China).

### 4.2. Animals and Treatment

SPF C57BL/6J male mice (18–20 g) were purchased from SinoBestBio (Beijing) Biotechnology Co., Ltd. (Beijing, China). (License No.: SCXK (Jing) 2019-0010; Certificate No.: 110324241101492711). The animals were housed in the Experimental Animal Center of Shanxi University of Chinese Medicine. The environmental temperature was maintained at 24–26 °C, and the animals were kept in standard laboratory animal cages. The relative humidity was at 55–65%, and the day–night rhythm ratio was at 1:1. The mice had free access to food and water. This experiment was approved by the Medical Ethics Committee of Shanxi University of Chinese Medicine (Approval No.: AWE202403228), and the experimental process was carried out in strict accordance with the guiding principles for animal research.

Emodin was prepared with a 0.5% CMC-Na solution to achieve the desired concentration before use and administered via oral gavage to mice at gradient doses of 40, 80, and 120 mg/kg. These dosage settings were determined based on commonly used protocols in recent in vivo pharmacological studies of emodin and preliminary experimental results, representing the low-, medium-, and high-gradient doses widely employed in this mouse model to facilitate observation of the dose-dependent pharmacodynamic patterns of emodin [40]. Acute toxicity studies have shown that the LD_50_ of emodin administered orally to mice is approximately 580 mg/kg. The highest dose used in this study (120 mg/kg) was significantly below the toxic threshold and remained within the safe tolerance range for mice. No significant acute toxicity or hepatic/renal impairment was observed within the administered dose range, ruling out interference from the drug’s intrinsic toxicity on experimental outcomes [41]. Additionally, according to the principle of equivalent dose conversion based on body surface area between humans and mice, the 40–120 mg/kg dose range in this study closely matches potential clinical therapeutic doses, aligning with the dose design principles for preclinical translational research of active ingredients in traditional Chinese medicine. This provides a reliable dosage reference for further mechanistic elucidation and pharmaceutical development of emodin [42] (to avoid ambiguity, the study group investigating the effects of emodin administration at doses of 40/80/120 mg/kg in normal healthy mice was designated as the sham group).

The mice were randomly assigned to one of two groups (experimental animals were randomly divided into groups using a computer-generated random number sequence): the sham group (*n* = 32, the mice were randomly assigned to four groups: normal control (NC), and normal + emodin 40/80/120 mg/kg (NE40/80/120)), and the MASLD group (*n* = 40, the mice were randomly assigned to five groups: normal control (NC), MASLD control (MC), and MASLD + emodin 40/80/120 mg/kg (ME40/80/120)). The normal control (NC) group received a standard diet, and the MASLD model was established via an 8-week high-fat MCD diet supplementation. All researchers involved in group allocation, animal treatment, outcome assessment, and data analysis were aware of the group allocation throughout the experiment.

### 4.3. Histological Observation of Liver and Colon Tissues

The fixed liver and colon tissues were sequentially processed through dehydration, clearing, paraffin embedding, and sectioning. Sections were stained with hematoxylin and eosin (H&E) to prepare pathological slides for observation. Histopathological changes were finally examined under a microscope.

### 4.4. Tissue Biochemical Assays

The levels of AST, ALT, HDL-C, LDL-C, TC, and TG in plasma were measured by using commercial kits. The collected liver and colon tissue samples were weighed, cut into small pieces, and transferred to a glass homogenizer. Nine volumes (*w*/*v*) of pre-cooled PBS (pH 7.4, 0.01 mol/L) were added, and the tissues were thoroughly ground to prepare homogenates. The homogenates were centrifuged (3000 r/min, 10 min), and the supernatant was collected for the BCA protein concentration assay.

### 4.5. Metabolomic Analysis

#### 4.5.1. Sample Preparation

Tissue homogenate: Take liver and colon tissues, add 9-fold volume of distilled water according to the ratio of weight (g): volume (mL) = 1:9. Cut the tissues into small pieces and fully grind them in a mortar to prepare 10% liver and colon tissue homogenates. Centrifuge at 4500 rpm for 10 min and collect the supernatant as the tissue homogenate.

Sample preparation: Take 100 μL of the tissue homogenate, add 4-fold volume of methanol, and vortex for 3 min to precipitate proteins as much as possible. Dry the sample under a nitrogen stream, re-dissolve it in 100 μL of methanol, centrifuge at 10,000 rpm for 10 min, and collect the supernatant as the serum sample. Take 10 μL of the supernatant from each of the above test samples and mix them thoroughly to prepare a quality control (QC) sample. During sample injection, insert one QC sample after each group of samples to evaluate the stability of the instrument.

#### 4.5.2. LC-MS Detection

Chromatographic conditions: UHPLC analysis of metabolites was conducted using an Ultimate 3000 UHPLC system (Thermo-Fisher Scientific, San Jose, CA, USA) equipped with an ACQUITY UPLC BEH C18 (2.1 × 100 mm, 1.7 μm), at a temperature of 40 °C. The mobile phase consisted of 0.1% (*v*/*v*) formic acid-H2O (B) and acetonitrile (A). The elution program was as follows: 5% (A) from 0 to 0.5 min, 10% (A) from 0.5 to 5 min, 15% (A) from 5 to 10 min, 25% (A) from 10 to 15 min, 35% (A) from 15 to 20 min, 60% (A) from 20 to 35 min, 100% (A) from 30 to 40 min, 100–5% (A) from 13 to 13.5 min, and 5% (A) from 13.5 to 16 min. A 5 μL injection volume and a flow rate of 0.3 mL/min were utilized.

Mass spectrometric conditions: An electrospray ionization source (ESI) was used with simultaneous positive and negative ion scanning modes. The spray voltage was 3.2 kV, the sheath gas flow rate was 40 arb, the auxiliary gas flow rate was 5 arb, the auxiliary gas heating temperature was 350 °C, the ion transfer tube temperature was 320 °C, the S-Lens RF Level was 50 V, the scanning range was 100–1000 *m*/*z*, and the collision energy was 30 eV.

#### 4.5.3. UHPLC/MS Data Processing

The Compound Discoverer 3.3 software was used for data pre-processing of LC-MS data, including peak deconvolution, peak alignment, peak calibration, and normalization. The normalized peak area data of each group were imported into SIMCA 14.1-P software for principal component analysis (PCA) and orthogonal partial least squares discriminant analysis (OPLS-DA) to evaluate the significant differences in lipid profiles among groups. The establishment of the OPLS-DA model was evaluated by R^2^Y, Q^2^, and the intercepts of R^2^ and Q^2^ to avoid overfitting. R^2^Y > 0.8, Q^2^ > 0.5, and both R^2^ and Q^2^ being close to 1.0 indicate a significant predictive ability of the model. The identification of the metabolites was further supported by referring to the retention times and mass spectrometric characteristics of standard substances, relevant literature, the METLIN metabolites database, Personal Compound Database and Library (PCDL, Agilent Technologies), the Human Metabolome Database (HMDB, https://hmdb.ca/, accessed on 12 May 2026), and the PubChem database (https://pubchem.ncbi.nlm.nih.gov/, accessed on 12 May 2026).

### 4.6. Preparation of Total DNA and High-Throughput Sequencing Analysis

Total microbial DNA was extracted from the colon content samples by the CTAB method, eluted with 50 μL of buffer, and stored at −80 °C. Subsequently, the V3–V4 hypervariable region of the bacterial 16S rDNA gene was amplified through thermocycling PCR, utilizing primers 341F (5′-CCTACGGGNGGCWGCAG-3′) and 805R (5′-GACTACHVGGGTATCTAATCC-3′). The PCR reaction program consisted of an initial denaturation step at 98 °C for 30 s, followed by 32 cycles, each cycle including denaturation at 98 °C for 10 s, annealing at 54 °C for 30 s, and extension at 72 °C for 45 s. The final extension was carried out at 72 °C for 10 min. The resultant PCR products were extracted using a 2% agarose gel and subsequently purified with AMPure XT magnetic beads. Quantification was ultimately achieved using Qubit.

Samples were assigned to paired-end sequences (reads) based on unique barcodes, and both the barcode and primer sequences were trimmed. The FLASH 1.2.11 software was applied to merge the paired-end sequences, while the fqtrim v0.94 software was utilized to process the original sequences in line with the pre-established filtering criteria, aiming to obtain high-quality available sequences. The Vsearch v2.3.4 software was employed to eliminate chimeric sequences, and the DADA2 1.38.0 software was used for the deduplication operation, thereby generating an ASV (Amplicon Sequence Variant) table.

Each ASV was compared with the SILVA database (http://www.arb-silva.de/, accessed on 12 May 2026) through the BLAST 2.17.0 tool, and taxonomic information was assigned to each ASV, thus forming an ASV table with taxonomic details. Based on this, the total abundance normalization method was adopted to normalize the counts of each species within each sample, converting them into relative abundances.

The R v4.1.2 software was utilized to conduct tests on the ASV abundance, taxonomic characteristics, diversity features, and metadata. The Kruskal–Wallis test and Dunn test multiple comparison methods were employed to analyze significant differences. The Kruskal–Wallis test and the Wilcoxon test were performed on all species. In combination with the linear discriminant analysis effect size (LEfSe), statistically significant marker species were sought, with the screening criteria being *p* < 0.05 (Benjamini–Hochberg correction) and LDA > 4.

### 4.7. Determination of SCFA Content in Colon Contents

A total of 50 mg of the colon content sample is transferred into a 2 mL grinding tube. Add a grinding bead and 500 μL of water (containing 0.5% phosphoric acid) into the tube. Then, grind the sample using a cryogenic grinder with the following operating parameters: 50 Hz for 3 min, repeated twice. Sonicate the sample in an ice-water bath for 30 min, let it stand at 4 °C for 30 min, and then centrifuge it (4 °C, 13,000× *g*, 15 min). Transfer the supernatant to a new 2 mL centrifuge tube. Pipette 200 μL of the supernatant, add 5 μL of the 2-ethylbutyric acid internal standard solution (prepared by dissolving 10 μL of the standard in 1 mL of ultrapure water), vortex to mix well, sonicate in an ice-water bath for 10 min, and centrifuge (4 °C, 13,000× *g*, and 10 min). Take the supernatant for GC-MS analysis. Take 10 μL of the supernatant from each of the above test samples and mix them thoroughly to prepare a quality control (QC) sample. During sample injection, insert one QC sample after each group of samples to evaluate the stability of the instrument.

Precisely pipette 25 μL of the 2-ethylbutyric acid standard into a 10 mL volumetric flask, and dilute it to the mark with ethyl acetate to obtain an internal standard stock solution with a concentration of 20 mM/L. Preparation of the mixed standard stock solution: Precisely pipette appropriate amounts of acetic acid, propionic acid, isobutyric acid, butyric acid, isovaleric acid, and valeric acid standards into a 10 mL volumetric flask, and dilute them to the mark with ethyl acetate to obtain a mixed standard stock solution with a final concentration of 200 mM/L for each of the above standards. Preparation of the mixed standard solution (containing the internal standard): Take 5 mL of the above mixed standard stock solution, add 1.67 mL of the internal standard stock solution, and mix thoroughly to obtain the mixed standard solution.

The separation was carried out on an Agilent HP FFAP capillary chromatographic column (30 m × 0.25 mm × 0.25 μm). Use high-purity helium (purity not less than 99.999%) as the carrier gas with a flow rate of 1.0 mL/min and an inlet temperature of 260 °C. The temperature programming is as follows: set the initial temperature of the column oven at 80 °C, then increase the temperature to 120 °C at a rate of 40 °C/min, increase it to 200 °C at a rate of 10 °C/min, and finally maintain the temperature at 230 °C for 3 min.

Mass spectrometric conditions: Use an electron impact ion source (EI) with an ion source temperature of 230 °C, a quadrupole temperature of 150 °C, a transfer line temperature of 230 °C, and an electron energy of 70 eV. Adopt the selective ion monitoring (SIM) scan mode.

### 4.8. Network Toxicology Study

The toxicity profile of emodin was predicted using the ADMETab platform (https://admetmesh.scbdd.com/, accessed on 12 May 2026). Potential target proteins of emodin were retrieved from the SwissTarget Prediction database (http://www.swisstargetprediction.ch/, accessed on 12 May 2026), the SEA Search Server (https://sea.bkslab.org, accessed on 12 May 2026/), and the ChEMBL database (https://www.ebi.ac.uk/chembl/, accessed on 12 May 2026). Targets related to liver injury were obtained by searching the Genecards database (https://www.genecards.org/, accessed on 15 May 2026), the OMIM database (https://omim.org/, accessed on 12 May 2026), and the TTD: Therapeutic Target Database (https://db.idrblab.net/ttd/, accessed on 12 May 2026) using the keyword “liver injury”. The intersection between emodin targets and liver injury-related targets was identified as a potential toxicity target.

The overlapping targets were imported into the DAVID database (https://davidbioinformatics.nih.gov/, accessed on 12 May 2026) for Gene Ontology (GO) and Kyoto Encyclopedia of Genes and Genomes (KEGG) pathway enrichment analyses. Bubble diagrams were generated using the bioinformatics online platform (http://www.bioinformatics.com.cn/, accessed on 12 May 2026). The selected targets were submitted to the STRING database to construct a protein–protein interaction (PPI) network. The resulting PPI network was then imported into Cytoscape 3.7.1 software to identify core therapeutic and toxicity-related targets.

### 4.9. Molecular Docking

The 3D structure of emodin was downloaded from the PubChem database. The crystal structures of key target proteins—BAX, BCL2, and ESR1—were retrieved from the RCSB PDB database (https://www.rcsb.org/, accessed on 12 May 2026), then dehydrated and hydrogenated using PyMOL 3.1.0 software. Molecular docking between emodin and the key targets was performed using AutoDock Vina 1.1.2. Docking poses with low binding energy were selected and visualized using PyMOL 3.1.0.

### 4.10. Western Blot Analysis

Approximately 30 mg of liver and colon tissues were collected and placed into 2 mL centrifuge tubes, followed by the addition of 500 μL lysis buffer to each specimen. Tissue samples were fully homogenized on ice using a tissue crusher and then centrifuged at 9500× *g* for 5 min. The protein concentration of the supernatant was determined using the Pierce BCA Protein Assay Kit according to the manufacturer’s instructions. Subsequently, 4 × loading buffer was added proportionally, and proteins were fully denatured by heating in a metal bath at 100 °C for 10 min. All samples were finally stored at −80 °C until use.

Protein samples were separated by SDS-PAGE and then electrotransferred onto PVDF membranes. The membranes were blocked with 5% skimmed milk at room temperature for 1 h. After blocking, membranes were incubated with primary antibodies against ESR1, BCL2, BAX, Occludin, Claudin-5, and the internal reference β-actin overnight at 4 °C. Following washing with TBST, membranes were incubated with horseradish peroxidase-conjugated secondary antibodies at room temperature. Protein bands were visualized using ECL chemiluminescence reagents, and band intensities were captured by an imaging system and quantitatively analyzed using ImageJ 1.53t/u software.

### 4.11. Statistical Analysis

Statistical analysis was performed using GraphPad Prism 10.0 software (Graphpad Software Inc., San Diego, CA, USA). Normality testing was conducted using the Shapiro–Wilk test, and homogeneity of variances was assessed with the Levene test. Measurement data conforming to normal distribution and homogeneity of variances were expressed as mean ± standard deviation. Overall comparisons among groups were performed using one-way ANOVA, followed by post hoc multiple comparisons using the Dunnett test; non-normal data were analyzed using the Kruskal–Wallis nonparametric test, with post hoc Dunn’s test for intergroup comparisons. Correlation analysis between two variables was conducted using the Spearman rank correlation coefficient. Based on standard sample size guidelines for in vivo pharmacological experiments and considering the degree of variation in this study, the sample size for each group was determined as *n* = 6 to ensure reliable statistical results. *p* < 0.05 was considered statistically significant, while *p* < 0.01 indicated highly significant differences.

Referring to the conventional sample size determination for similar in vivo pharmacological experiments and considering the degree of variation in this study, the initial number of mice per group was set at 8. During the experiment, 2 mice that failed to establish models, exhibited abnormal individual conditions, or failed sample collection and testing were excluded. Ultimately, 6 valid samples per group were included for statistical analysis.

## 5. Conclusions

**Figure 10:**
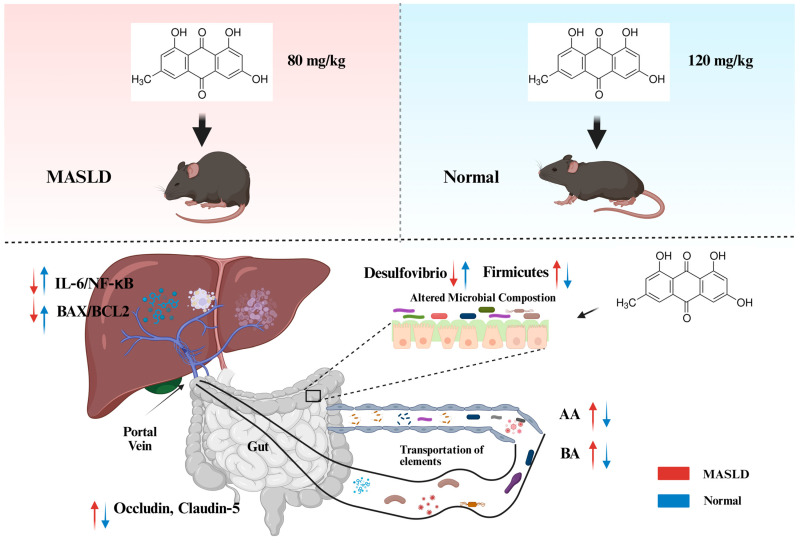
Schematic diagram of the proposed mechanism.

This study investigated the dose-dependent effects of emodin on the gut–liver axis and intestinal barrier via multi-dimensional experiments. In the MASLD model, low and medium doses (40–80 mg/kg) of emodin alleviated hepatic steatosis and inflammation, repaired colonic tissue damage, decreased serum ALT and AST levels, inhibited the expression of hepatic inflammatory factors such as IL-6 and NF-κB, remodeled gut microbiota composition, and elevated colonic SCFA production. Meanwhile, emodin positively modulated the expression of colonic tight junction proteins (Occludin, Claudin-5), hepatic ESR1, BCL2, and BAX. The dose of 80 mg/kg was identified as the optimal concentration for emodin to exert protective effects. A high dose of emodin (120 mg/kg) exhibited toxic performance: it not only weakened the colonic repair effect in MASLD mice but also induced obvious gut and liver toxicity in normal mice, presenting as hepatocyte injury, exacerbated inflammation, gut microbiota dysbiosis, reduced SCFA content, and impaired intestinal barrier integrity. Moreover, abnormal metabolomic characteristics were observed, and divergent metabolic disorder pathways existed between MASLD and normal mice. Correlation analysis showed that gut microbiota structure, SCFA content, and the expression of ESR1, BCL2, and BAX displayed synergistic variation trends (Figure 10).

In summary, emodin exerts dose-dependent effects on the gut–liver system: low-to-medium doses produce protective effects, while high doses induce toxicity, with the optimal dose around 80 mg/kg. The biological actions of emodin are closely linked to the gut microbiota–SCFA–gut–liver axis network.

## Figures and Tables

**Table 1:** Molecular binding capacity.

Compound	Target	PDB ID	Minimum Binding Energy (kcal/mol)
Emodin	BAX	4s0o	−8.5
	BCL2	8hts	−7.7
	ESR1	7baa	−7.9

## Data Availability

Data will be made available on request.

## Supplementary Materials

The following supporting information can be downloaded at: https://www.mdpi.com/article/10.3390/ijms27104411/s1.

## Abbreviations

The following abbreviations are used in this manuscript:

MASLD	Metabolic dysfunction-associated steatotic liver disease
MCD	Methionine and choline-deficient
TC	Total cholesterol
TG	Triglyceride
PCA	Principal component analysis
OPLS-DA	Orthogonal partial least squares discriminant analysis
AA	Acetic acid
PA	Propionic acid
IBA	Isobutyric acid
BA	Butyric acid
IVA	Isovaleric acid
VA	Valeric acid
4-MVA	4-methylvaleric acid
HA	Hexanoic acid

## References

[1] Riazi K., Azhari H., Charette J.H., Underwood F.E., King J.A., Afshar E.E., Swain M.G., Congly S.E., Kaplan G.G., Shaheen A.-A. (2022). The prevalence and incidence of NAFLD worldwide: A systematic review and meta-analysis. Lancet Gastroenterol. Hepatol..

[2] Younossi Z.M., de Avila L., Petta S., Hagström H., Kim S.U., Nakajima A., Crespo J., Castera L., Alkhouri N., Zheng M.-H. (2025). Predictors of fibrosis, clinical events and mortality in MASLD: Data from the Global-MASLD study. Hepatology.

[3] Younossi Z.M., de Avila L., Petta S., Hagström H., Kim S.U., Nakajima A., Crespo J., Castera L., Alkhouri N., Zheng M.-H. (2025). Global performance of non-invasive tests in MASLD: Insights from the G-MASLD study. Hepatology.

[4] Younossi Z.M., Zelber-Sagi S., Lazarus J.V., Wong V.W.-S., Yilmaz Y., Duseja A., Eguchi Y., Castera L., Pessoa M.G., Oliveira C.P. (2025). Global Consensus Recommendations for Metabolic Dysfunction-Associated Steatotic Liver Disease and Steatohepatitis. Gastroenterology.

[5] Balakrishnan M., Rehm J. (2024). A public health perspective on mitigating the global burden of chronic liver disease. Hepatology.

[6] Zhou X.D., Fan Q.Y., Byrne C.D., Targher G., Muthiah M.D., Huang D.Q., Chen Q.-F., Noureddin M., Li W., Ratziu V. (2025). Combination therapies for metabolic dysfunction-associated steatohepatitis: Challenges and opportunities. Gut.

[7] Yuan Y., Gong M., Pan R., Shi X., Li X., Huang H., Zhao J. (2025). Artificially fermented dark loose tea ameliorates metabolic-associated fatty liver disease by activating the PI3K/AKT signalling pathway and regulating gut microbiota dysbiosis. Food Chem. X.

[8] Ma C., Wang J., Song X., Wang X., Zong S. (2025). Molecular mechanisms and clinical applications of gut microbiota-derived bioactive compounds in metabolic dysfunction-associated fatty liver disease. Front. Immunol..

[9] Yang Q., Cai X., Li S., Xiao Z. (2026). Association Between Nonalcoholic Fatty Liver Disease and the Dietary Index for Gut Microbiota: A Cross-Sectional Study. Food Sci. Nutr..

[10] Cheng X., Li S., Huang C., Yang Y., Liu R., Cao S., Luo L., Lu B. (2026). Targeting the intestinal barrier with traditional Chinese medicine for non-alcoholic fatty liver disease: Mechanistic insights and therapeutic perspectives. Chin. Med..

[11] Jiang S., Lu C., Zhao C., Yuan F., Jin J., Wang Y., Wang X., Yan T., Gao T. (2025). Pectin Polysaccharides from Cascara Prevent Metabolic Dysfunction-Associated Fatty Liver Disease Development by Regulating Gut Microbiota and Liver Metabolism. J. Agric. Food Chem..

[12] Hong Y., Yang J., Wang Y., Chen D., Wu A., Li M., Ou W., Lin G., Lin C., Liang Y. (2025). Modulation of Gut Microbes and Hepatic Metabolites by PCP Ameliorates NASH and Fatigue-like Performance in Mice. Nutrients.

[13] Liu M., Wang Y., Huang H. (2026). Gut Microbiota-Derived Propionic Acid Mediates ApoA-I-Induced Amelioration of MASLD via Activation of GPR43-Ca^2+^-CAMKII-ATGL Hepatic Lipolysis. Int. J. Mol. Sci..

[14] Li J., Hu W., Li C., Tang H., Kuang H., Wang M. (2026). Recent advances in hawthorn (*Crataegus* L.) polysaccharides for prevention and treatment of non-alcoholic fatty liver disease (NAFLD): A review. Int. J. Biol. Macromol..

[15] Semwal R.B., Semwal D.K., Combrinck S., Viljoen A. (2021). Emodin—A natural anthraquinone derivative with diverse pharmacological activities. Phytochemistry.

[16] Zhang Q., Chen W.W., Sun X., Qian D., Tang D.D., Zhang L.L., Li M.Y., Wang L.Y., Wu C.-J., Peng W. (2022). The versatile emodin: A natural easily acquired anthraquinone possesses promising anticancer properties against a variety of cancers. Int. J. Biol. Sci..

[17] Ding Z., Zheng M., Mou B., Li Y., Qin B., Qiu L., Yang X., Ren Y. (2026). Emodin induces oxidative stress and Ferroptosis in hepatocellular carcinoma cells through the miR-4465/NFE2L3/HMGCR/GPX4 signaling Axis. Life Sci..

[18] Chen L., Liang B., Xia S., Wang F., Li Z., Shao J., Zhang Z., Chen A., Zheng S., Zhang F. (2023). Emodin promotes hepatic stellate cell senescence and alleviates liver fibrosis via a nuclear receptor (Nur77)-mediated epigenetic regulation of glutaminase 1. Br. J. Pharmacol..

[19] Huang C., Zhang Y., Xu Y., Wei S., Yang T., Wang S., Li C., Lin H., Li X., Zhao S. (2024). Prepared Radix Polygoni Multiflori and emodin alleviate lipid droplet accumulation in nonalcoholic fatty liver disease through MAPK signaling pathway inhibition. Aging.

[20] Guo C., Huang Q., Wang Y., Yao Y., Li J., Chen J., Wu M., Zhang Z., Mingyao E., Qi H. (2023). Therapeutic application of natural products: NAD+ metabolism as potential target. Phytomedicine.

[21] Li M., Wang Y.R., Wang X., Xiao X.-L., Sun Y.-H., Zhang S.-A., Dang Y.-Q., Wang K., Zhou W.-J. (2025). Emodin Enhances Rosiglitazone’s Therapeutic Profile by Dual Modulation of SREBP1-Mediated Adipogenesis and PPARγ-Driven Thermogenesis. Pharmaceuticals.

[22] Li Z., Gu Y., Yang J., Wang S., Li S., Chen P., Yao R., Liu F., Wang Y., Wang R. (2025). Emodin and physcion alleviate cholestatic liver injury by targeting FXR: Hepatoprotective components identified in processed Polygonum multiflorum Thunb. using a comprehensive two-dimensional biochromatography system. Front. Pharmacol..

[23] Li Y., Duan S., Zhang Y., Liu R., Sun R., Wu J., Ma Z., Li X. (2025). Precise subcellular organelle-targeted analyses of hepatotoxicity of Polygonum multiflorum. Chin. Herb. Med..

[24] Wu L., Chen S., Yang Q., Li X., Liu L., Ji S., Tang S., Zhang S., Wang Y., Chen R. (2026). Cytochrome P450-mediated detoxification and NF-κB/NLRP3 pathway-driven hepatotoxicity of emodin: Multiomics and laboratory evidence. Chem. Biol. Interact..

[25] Hu S., Guo X., Tu L., Xiong H., Lu X., Xu X., Li Y., Yu Y., Zhou C., Hui K. (2025). The efficacy and toxicity equilibrium of emodin for liver injury: A bidirectional meta-analysis and machine learning. Phytomedicine.

[26] Xia X., He X., Huang J., Hou X., Lin C., Liu Y., Liu M. (2024). Emodin induced hepatic steatosis in BALb/c mice by modulating the gut microbiota composition and fatty acid metabolism. Front. Pharmacol..

[27] Rong Z., Li B., Liu C., Liao L. (2026). Paeoniflorin attenuates sepsis-induced liver injury by reprogramming macrophage polarization via the TLR4/NF-κB pathway. Front. Immunol..

[28] Sharma R., Tiku A.B. (2016). Emodin inhibits splenocyte proliferation and inflammation by modulating cytokine responses in a mouse model system. J. Immunotoxicol..

[29] Liao H., Zhang J., Zhang J., Chen Y., Chen X., Meng Y., Chen J. (2026). Immune cells in liver injury: From pathogenic mechanisms to immunotherapy. Front. Immunol..

[30] Tan X., Li Q., Ma Z., Sun L., Zhao X., Chen J., Wang J., Weng X., Chen L., Chen Z. (2026). CD1d functions as a ligand for PIRA2 to drive macrophage activation in nonalcoholic fatty liver disease. Cell Death Dis..

[31] Piseddu I., Jochheim L.S., Boettcher K., Scheiner B., Sinner F., Gairing S.J., Thaler M., Enssle S., Karin M., Zarka V. (2025). Early mortality in atezolizumab/bevacizumab for HCC is associated with impaired liver function and alterations of systemic immunity. JHEP Rep..

[32] Huang Y., Chen D., Chen Y., Chen F., Chen Y., Huang Y. (2026). Punicalagin Alleviates Acute Liver Injury via Dual STAT1/NF-κB Inhibition and STAT3 Activation to Orchestrate M1-to-M2 Macrophage Polarization. Phytother. Res..

[33] Krautkramer K.A., Fan J., Bäckhed F. (2021). Gut microbial metabolites as multi-kingdom intermediates. Nat. Rev. Microbiol..

[34] Ping Y., Shan J., Qin H., Li F., Qu J., Guo R., Han D., Jing W., Liu Y., Liu J. (2024). PD-1 signaling limits expression of phospholipid phosphatase 1 and promotes intratumoral CD8+ T cell ferroptosis. Immunity.

[35] Li Y., Wang J., Wang H., Ma X., Ren D., Wang B. (2026). A novel exopolysaccharide from Lactiplantibacillus plantarum H6 improves cholesterol metabolism via Muribaculum-mediated activation of the enterohepatic FXR-FGF15 axis. Gut Microbes.

[36] Wang J., Zhu N., Su X., Gao Y., Yang R. (2023). Gut-Microbiota-Derived Metabolites Maintain Gut and Systemic Immune Homeostasis. Cells.

[37] Su X., Gao Y., Yang R. (2022). Gut Microbiota-Derived Tryptophan Metabolites Maintain Gut and Systemic Homeostasis. Cells.

[38] Schoeller A., Karki K., Jayaraman A., Chapkin R.S., Safe S. (2022). Short chain fatty acids exhibit selective estrogen receptor downregulator (SERD) activity in breast cancer. Am. J. Cancer Res..

[39] Liu H., Cao M., Jin Y., Jia B., Wang L., Dong M., Han L., Abankwah J., Liu J., Zhou T. (2023). Network pharmacology and experimental validation to elucidate the pharmacological mechanisms of Bushen Huashi decoction against kidney stones. Front. Endocrinol..

[40] Du D., Lou Y., Zhou L., Tian J., Zhang T., Qiu Z., Rong X. (2026). Emodin Attenuates Rheumatoid Arthritis by Modulating the NF-κB/HIF-1α/VEGF Signaling Pathway. Int. J. Mol. Sci..

[41] Lei X., Chen G., Chen K.L., Li J. (2008). Acute toxicity study of emodin in mice. Pharmacol. Clin. Chin. Mater. Medica.

[42] National Toxicology Program (2001). NTP Toxicology and Carcinogenesis Studies of EMODIN (CAS NO. 518-82-1) Feed Studies in F344/N Rats and B6C3F1 Mice. Natl. Toxicol. Program Tech. Rep. Ser..

