# Supplementary material for: Emodin Exerts Dual Hepatoprotective/Hepatotoxic Effects Dependent on Metabolic Microenvironment via Gut–Liver Axis Crosstalk: A Multi-Omics Study in MAFLD and Normal Mice
Source: Int J Mol Sci. 2026 May 15;27(10):4411. doi: 10.3390/ijms27104411 (PMC13207176; doi:10.3390/ijms27104411)
Supplement: Supplementary file 1 [file ijms-27-04411-s001.zip › ijms-4281808-supplementary.pdf]

Supporting Information

Emodin exerts dual hepatoprotective/hepatotoxic effects dependent on metabolic microenvironment via gut-liver axis crosstalk: A multi-omics study in MAFLD and normal mice

Table S1 22 different metabolites in the liver tissues of mice in the MASLD group

Name	Formula	m/z	RT [min]	Reference Ion	HMDB
Histidylglycine	$C_8H_{12}N_4O_3$	213.0982	0.813	$[M+H]^{+1}$	HMDB0028885
Phosphorylcholine	$C_5H_{14}NO_4P$	184.0732	0.916	$[M+K]^{+1}$	HMDB0001565
Glycerophosphoglycerol	$C_6H_{15}O_8P$	245.0429	0.929	$[M-H]^{-1}$	HMDB0240316
Dihydrothymine	$C_5H_8N_2O_2$	129.0660	0.964	$[M+H]^{+1}$	HMDB0000079
5-Methylcytosine	$C_5H_7N_3O$	126.0663	0.978	$[M+H]^{+1}$	HMDB0002894
Thiophanate-methyl	$C_{12}H_{14}N_4O_4S_2$	341.0391	0.999	$[M-H]^{-1}$	HMDB0259025
Hypoxanthine	$C_5H_4N_4O$	137.0458	1.001	$[M+H]^{+1}$	HMDB0000157
Uridine	$C_9H_{12}N_2O_6$	243.0620	1.007	$[M-H]^{-1}$	HMDB0000296
Deoxyuridine	$C_9H_{12}N_2O_5$	227.0668	1.193	$[M-H]^{-1}$	HMDB0000012
Inosine	$C_{10}H_{12}N_4O_5$	267.0736	1.203	$[M-H]^{-1}$	HMDB0000195
(1-Ribosylimidazole)-4-acetate	$C_{10}H_{14}N_2O_6$	257.0779	1.304	$[M-H]^{-1}$	HMDB0002331
Thymidine	$C_{10}H_{14}N_2O_5$	241.0828	1.576	$[M-H]^{-1}$	HMDB0000273
Nicotianamine	$C_{12}H_{21}N_3O_6$	302.1359	2.854	$[M-H]^{-1}$	HMDB0255025
Marimastat	$C_{15}H_{29}N_3O_5$	330.2037	3.405	$[M-H]^{-1}$	HMDB0014924
Isopentenyladenosine	$C_{15}H_{21}N_5O_4$	336.1664	4.188	$[M+H]^{+1}$	HMDB0304396
MG(0:0/20:5(5Z,8Z,11Z,14Z,17Z)/0:0)	$C_{23}H_{36}O_4$	377.2683	6.062	$[M+H]^{+1}$	HMDB0011550
Dehydrophytosphingosine	$C_{18}H_{37}NO_3$	298.2739	9.66	$[M+H-H2O]^{+1}$	HMDB0038057
Palmitoleoyl-EA	$C_{18}H_{35}NO_2$	298.2740	9.672	$[M+H]^{+1}$	HMDB0013648
11-cis-retinal	$C_{20}H_{28}O$	285.2211	10.585	$[M+H]^{+1}$	HMDB0001358
MG(0:0/22:6(4Z,7Z,10Z,13Z,16Z,19Z)/0:0)	$C_{25}H_{38}O_4$	403.2840	13.28	$[M+H]^{+1}$	HMDB0011557
2-Arachidonoylglycerol	$C_{23}H_{38}O_4$	379.2837	13.361	$[M+H]^{+1}$	HMDB0004666
PG(16:0/22:6(4Z,7Z,10Z,13Z,16Z,19Z))	$C_{44}H_{75}O_{10}P$	793.5034	15.656	$[M-H]^{-1}$	HMDB0010584

Table S2 16 different metabolites in the colon tissues of mice in the MASLD group

Name	Formula	m/z	RT [min]	Reference Ion	HMDB
Sedoheptulose	$C_7H_{14}O_7$	209.066	0.916	$[M-H]^{-1}$	HMDB0003219
7-Aminomethyl-7-carbaguanine	$C_6H_{13}NO_5$	180.08799	1.216	$[M+H]^{+1}$	HMDB0011690
7-Methyluric acid	$C_6H_6N_4O_3$	181.03579	1.303	$[M-H]^{-1}$	HMDB0011107
Hydroxyphenylacetylglycine	$C_{10}H_{11}NO_4$	208.0609	4.019	$[M-H]^{-1}$	HMDB0000735
Lovastatin acid	$C_{24}H_{38}O_6$	423.27394	5.288	$[M+H]^{+1}$	HMDB0254177
Crocin 4	$C_{24}H_{42}O_7P_2$	503.2325	5.766	$[M-H]^{-1}$	HMDB0039122
Dodecanedioic acid	$C_{12}H_{22}O_4$	229.14405	6.786	$[M-H]^{-1}$	HMDB0000623
12S-HHT	$C_{17}H_{28}O_3$	279.19657	9.417	$[M-H]^{-1}$	HMDB0012535
9,10,13-TriHOME	$C_{18}H_{34}O_5$	331.24754	10.024	$[M+H]^{+1}$	HMDB0004710
Endomorphin-1	$C_{27}H_{54}N_4OS_5$	306.1515	11.085	$[M+2H]^{+2}$	HMDB0005773
11,12-Epoxyeicosatrienoic acid	$C_{14}H_{33}N_4O_2P$	321.24216	11.854	$[M+H]^{+1}$	HMDB0004673
Anandamide	$C_{22}H_{37}NO_2$	348.28939	12.573	$[M+H]^{+1}$	HMDB0004080
Benzoyl ecgonine	$C_{16}H_{19}NO_4$	290.13853	12.985	$[M+H]^{+1}$	HMDB0041836
Isopimaric acid	$C_{13}H_{30}N_6S$	303.23163	13.438	$[M+H]^{+1}$	HMDB0036811
Palmitoylethanolamide	$C_{18}H_{37}NO_2$	300.28938	13.499	$[M+H]^{+1}$	HMDB0002100
4-Acetamido-2-amino-6-nitrotoluene	$C_9H_{11}N_3O_3$	210.08713	15.605	$[M+H]^{+1}$	HMDB0060384

Table S3 17 different metabolites in the liver tissues of normal mice

Name	Formula	m/z	RT [min]	Reference Ion	HMDB ID
Bis(2-ethylhexyl)adipate	$C_{22}H_{42}O_4$	371.31556	11.25	$[M+H]^{+1}$	HMDB0040270
PC(O-16:1(11Z)/2:0)	$C_{26}H_{52}NO_7P$	522.35529	11.065	$[M+H]^{+1}$	HMDB0062195
3,5,7-Octatriyn-1-ol	C_8H_6O	136.07583	1.123	$[M+NH4]^{+1}$	HMDB0038998
PC(20:3(8Z,11Z,14Z)/0:0)	$C_{28}H_{52}NO_7P$	546.35551	10.555	$[M+H]^{+1}$	HMDB0010394
Ergothioneine;Thiolhistidine-betaine	$C_9H_{15}N_3O_2S$	230.09582	0.925	$[M+H]^{+1}$	HMDB0003045
sn-glycero-3-Phosphocholine	$C_8H_{20}NO_6P$	258.11008	1.027	$[M+H]^{+1}$	HMDB0000086
PE(22:6(4Z,7Z,10Z,13Z,16Z,19Z)/0:0)	$C_{27}H_{44}$ NO_7P	524.27876	10.276	$[M-H]^{-1}$	HMDB0011526
PC(18:3(9Z,12Z,15Z)/0:0)	$C_{26}H_{48}$ NO_7P	518.32427	9.285	$[M+H]^{+1}$	HMDB0010388
PC(22:5(4Z,7Z,10Z,13Z,16Z)/0:0)	$C_{30}H_{52}NO_7P$	570.3555	10.317	$[M+H]^{+1}$	HMDB0010402
isodesmosine	$C_{24}H_{40}N_5O_8$	527.29606	10.312	$[M+H]^{+1}$	HMDB0000739
PC(20:5(5Z,8Z,11Z,14Z,17Z)/0:0)	$C_{28}H_{48}NO_7P$	542.32424	9.11	$[M+H]^{+1}$	HMDB0010397
Sativic acid	$C_{18}H_{36}O_6$	331.24773	9.939	$[M+H-H_2O]^{+1}$	HMDB0302836
9_10_13-trihydroxy-11-octadecenoicacid	$C_{18}H_{34}O_5$	331.24763	9.951	$[M+H]^{+1}$	HMDB0004710
Adenosine	$C_{10}H_{13}N_5O_4$	268.10398	1.178	$[M+H]^{+1}$	HMDB0000050
PE(0:0/22:5(4Z,7Z,10Z,13Z,16Z))	$C_{27}H_{46}NO_7P$	526.29525	10.262	$[M-H]^{-1}$	HMDB0011494
PC(22:4(7Z,10Z,13Z,16Z)/0:0)	C_{30} $H_{54}NO_7P$	572.37131	11.262	$[M+H]^{+1}$	HMDB0010401

Table S4 21 different metabolites in the colon tissues of normal mice

Name	Formula	m/z	RT [min]	Reference Ion	HMDB ID
Hypoxanthine	$C_5H_4N_4O$	137.04583	0.99	$[M+H]^{+1}$	HMDB0000157
L-Norleucine	$C_6H_{13}NO_2$	132.10202	1.254	$[M+H]^{+1}$	HMDB0001645
Choline	$C_5H_{13}NO$	104.10741	0.872	$[M+H]^{+1}$	HMDB0000097
5-Aminovaleric acid	$C_5H_{11}NO_2$	118.08656	1.166	$[M+H]^{+1}$	HMDB0003355
Xanthine	$C_5H_4N_4O_2$	151.02496	1.178	$[M-H]^{-1}$	HMDB0000292
Proline	$C_5\ H_9\ N\ O_2$	116.07093	0.915	$[M+H]^{+1}$	HMDB0000162
Uric acid	$C_5H_4N_4O_3$	167.02	0.96	$[M-H]^{-1}$	HMDB0000289
D-Erythrose 4-phosphate	$C_4H_9O_7P$	199.00055	0.965	$[M-H]^{-1}$	HMDB0001321
alpha-methylstyrene	C_9H_{10}	119.08579	8.79	$[M+H]^{+1}$	HMDB0059899
Pantothenate	$C_9H_{17}NO_5$	220.11805	2.505	$[M+H]^{+1}$	HMDB0000210
Spermine	$C_{10}H_{26}N_4$	203.22321	0.779	$[M+H]^{+1}$	HMDB0001256
N-Acetylneuraminic acid	$C_{11}H_{19}NO_9$	308.09903	0.917	$[M-H]^{-1}$	HMDB0000230
I-Urobilinogen	$C_{33}H_{44}N_4O_6$	593.33329	5.217	$[M+H]^{+1}$	HMDB0001898
Palmitoyl Serinol	$C_{19}H_{39}NO_3$	330.30011	10.199	$[M+H]^{+1}$	HMDB0013654
Azelaic acid	$C_9H_{16}O_4$	187.09671	4.741	$[M-H]^{-1}$	HMDB0000784
Adipic acid	$C_6H_{10}O_4$	145.04947	1.394	$[M-H]^{-1}$	HMDB0000448
L-Phenylalanine	$C_9H_{11}NO_2$	149.05986	2.257	$[M+H]^{+1}$	HMDB0000159
PGB2	$C_{20}H_{30}O_4$	335.22139	5.509	$[M+H]^{+1}$	HMDB0004236
6-Hydroxypentadecanedioic acid	$C_{15}H_{28}O_5$	287.18678	3.689	$[M-H]^{-1}$	HMDB0031885
15-deoxy-delta-12,14-PGJ2	$C_{20}H_{28}O_3$	317.21094	5.51	$[M+H]^{+1}$	HMDB0005079
fluorochloridone	$C_{12}H_{10}Cl_2F_3NO$	310.00241	2.225	$[M-H]^{-1}$	HMDB0252372
